# Management of Anticoagulation during Extracorporeal Membrane Oxygenation in Children

**DOI:** 10.3390/pediatric14030039

**Published:** 2022-07-11

**Authors:** Madhuradhar Chegondi, Niranjan Vijayakumar, Balagangadhar R. Totapally

**Affiliations:** 1 Division of Pediatric Critical Care Medicine, Stead Family Children’s Hospital, University of Iowa, Iowa City, IA 52242, USA; 2 Department of Pediatrics, Carver College of Medicine, University of Iowa, Iowa City, IA 52242, USA; 3 Division of Cardiac Critical Care, Boston Children’s Hospital, Harvard Medical School, Boston, MA 02115, USA; niranjan.vijayakumar@childrens.harvard.edu; 4 Division of Critical Care Medicine, Nicklaus Children’s Hospital, Miami, FL 33155, USA; balagangadhar.totapally@nicklaushealth.org; 5 Herbert Wertheim College of Medicine, Florida International University, Miami, FL 33199, USA

**Keywords:** extracorporeal membrane oxygenation, ECMO, extracorporeal life support, anticoagulation, bleeding, thrombosis, children

## Abstract

Extracorporeal Membrane Oxygenation (ECMO) is often used in critically ill children with severe cardiopulmonary failure. Worldwide, about 3600 children are supported by ECMO each year, with an increase of 10% in cases per year. Although anticoagulation is necessary to prevent circuit thrombosis during ECMO support, bleeding and thrombosis are associated with significantly increased mortality risk. In addition, maintaining balanced hemostasis is a challenging task during ECMO support. While heparin is a standard anticoagulation therapy in ECMO, recently, newer anticoagulant agents are also in use. Currently, there is a wide variation in anticoagulation management and diagnostic monitoring in children receiving ECMO. This review intends to describe the pathophysiology of coagulation during ECMO support, review of literature on current and newer anticoagulant agents, and outline various diagnostic tests used for anticoagulation monitoring. We will also discuss knowledge gaps and future areas of research.

## 1. Introduction

During extracorporeal membrane oxygenation (ECMO), the patient blood is continuously exposed to the ECMO circuit tubing and membrane oxygenator. The coagulation cascade is activated when the blood comes in contact with any non-endothelial surface [1,2]. The vascular endothelium plays a pivotal role in maintaining a balance between procoagulant and anticoagulant activity by mediating interactions between various blood cells with plasma factors resulting in uninterrupted blood flow in the body. During extracorporeal circulation, procoagulant activity is predominant, thus leading to thrombosis and occlusion of vascular cannulas and circuits [1]. Exogenous anticoagulant therapy is required to prevent procoagulant activity and thrombosis in the patient as well as in the extracorporeal circuit. However, systemic bleeding can occur with anticoagulation therapy. During the ECMO, significant complications can occur due to thrombosis and bleeding, which mandates close anticoagulation monitoring.

## 2. Coagulation and Fibrinolysis Cascades

Hemostasis starts at the site of vascular injury. The disrupted vascular endothelium releases von Wiliebrand factor and tissue factor, which exerts procoagulant properties and helps in platelet plug formation [3]. The cell surface glycoprotein receptors help platelets adhesion to the endothelial cells and the extracellular matrix. Following adhesion, platelets get activated through their secreted substances and intracellular signaling mechanisms [4]. The extrinsic pathway of coagulation starts when the tissue factor binds to factor VII, which further activates factor X with the help of platelet-derived phospholipid and calcium. The activated factor X interacts with the factor V, phospholipid, and calcium and converts prothrombin to thrombin (factor IIa). Using a positive feedback loop, thrombin activates the intrinsic pathway coagulation factors V, VIII, XI, and XIII, and further produces thrombin. Prothrombin to thrombin conversion represents the initial step in the common pathway. Thrombin further activates fibrinogen to form fibrin strands. Then factor XIII converts the fibrin strands into fibrin mesh, which ultimately stabilizes the platelet plug [4].

Fibrinolysis, similar to coagulation cascade, is a highly regulated pathway that dissolves the thrombus and restores the blood flow. The initial step involves the plasminogen to plasmin conversion through the tissue and urokinase plasminogen activators (tPA/uPA). Then the plasmin breaks down the fibrin into fibrin degradation products. The fibrinolysis pathway is mainly inhibited by the plasminogen activator inhibitor (PAI) and thrombin-activatable fibrinolysis inhibitor (TAFI) [5].

## 3. Coagulation System in Children

The coagulation system maturation continues postnatally, and most coagulation protein levels reach adult levels by six months of age. In addition, during the neonatal period, AT levels are less than 50% of adult levels. However, the risk of thrombosis is less and at the baseline, there is a higher risk of bleeding in children than in adults due to unclear mechanisms [6]. Similarly, the hemodilution effect during the ECMO circuit priming is much higher in children resulting significant decrease in platelet count and coagulation factors [7].

## 4. The Need for Anticoagulation during ECMO

Surgical incision and the inflammatory state of critical illness in children receiving ECMO predispose them to a procoagulant state. Low flow areas and shearing forces during turbulence in the ECMO circuit can lead to thrombosis. A complex inflammatory response occurs when blood is exposed to artificial surfaces like an extracorporeal circuit [2]. ECMO activates this inflammatory response mediated by multiple blood cells and plasma proteins. Platelet activation occurs with adhesion to the extracorporeal circuit, leading to platelet aggregation and coagulation cascade activation [1,8]. Platelet activation and consumption are continuous while a patient is on the ECMO. The intrinsic coagulation pathway predominantly mediates the coagulation during ECMO, except in patients with recent surgery, where the extrinsic coagulation pathway has an important role. Activated monocytes release tissue factors into the extracorporeal system and activate the extrinsic coagulation pathway. Systemic anticoagulation is an essential part of ECMO therapy in children. Although unfractionated heparin is the most commonly used anticoagulant, recently, there is increased use of direct thrombin inhibitors for anticoagulation during ECMO therapy.

**Figure 1:**
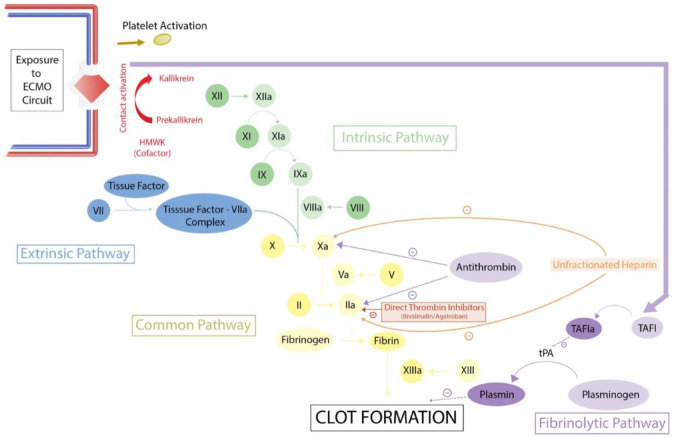
Coagulation & Fibrinolytic pathways.

Elevation of factor XIIa and fibrin degradation products following ECMO initiation indicates the activation of contact and fibrinolytic pathways, as shown in Figure 1 [9]. During ECMO, the alternative complement pathway is mainly activated. Activation of these multiple pathways results in consumptive coagulopathy with a decline in platelet count and coagulation factors deficiency [10,11].

## 5. Anticoagulation Management with Heparin

Anticoagulation therapy is much needed to avoid clotting during ECMO support. Unfractionated heparin (UFH) is the anticoagulant of choice during ECMO [12,13,14]. Mechanism of action of heparin involves inhibition of thrombin and factor Xa via activation and binding of antithrombin (AT) [15]. AT is a natural anticoagulant, which binds at the specific pentasaccharide sequence of heparin and results in 1000-folds inhibition of thrombin [16]. Heparin does not affect thrombin generation or fibrin-bound thrombin; it acts only on already formed thrombin. UFH is a much more efficient inactivator of thrombin due to its molecule size compared to LMWH. In addition, heparin has a short half-life, so its effect is instantaneous; however, heparin bioavailability may be affected by the binding of coagulation factors and proteins secreted by platelets and endothelial cells [17]. Heparin can be infused into the patient or the ECMO circuit.

At the time of ECMO cannulation, a bolus dose of heparin is given. The dose is between 50–100 units per kg body weight. A lower dose should be considered in patients with preexisting bleeding, recent surgery, or cardiopulmonary bypass. Based on data from cardiopulmonary bypass, traditionally activated clotting time (ACT) has been followed to guide the efficacy of anticoagulation on the circuit. The usual target ACT in the patient at the time of cannulation is above 300 s. During circuit priming, 100 units per kg boluses are given into the ECMO circuit to target circuit ACT above 400 s. Following the bolus dose of heparin, frequent monitoring of ACT is necessary, and once the ACT drops to 300 s or lower, heparin infusion is initiated. Heparin infusion is usually started at 10–20 units per kg per h. While weaning off ECMO, the heparin infusion rate should be increased by 10% to keep the ACT values at the upper end of the accepted range. Additional heparin boluses should be used to achieve the target ACT parameters. Depending on center experience and the type of equipment used, the standard ACT parameters range varies from center to center [18].

ACT poorly correlates with the heparin concentration and is not approved for monitoring direct thrombin inhibitors (DTIs) therapy [12]. As a result, the recent ECMO guidelines have suggested aPTT and/or anti-factor Xa levels as a better guide to monitoring UFH efficacy [12,19]. Depending on center practices, aPTT levels of 60–80 s or Anti-Xa levels of 0.3–0.7 units/mL have been suggested for titration of UFH infusion rate [12,13]. A recent survey among the pediatric ECMO centers in the USA reported anti-factor Xa assay as the most commonly used test, followed by ACT [13].

Since the anticoagulant activity of heparin depends on the circulating AT levels, checking AT levels may be helpful in patients with high heparin requirements [20]. In patients with low concentrations of AT, fresh frozen plasma (FFP) and recombinant AT concentrate can be used [21]. Compared to FFP, recombinant AT concentrate is better at achieving desired AT levels [22]. A recent RCT has demonstrated that the AT administration at AT levels of less than 60% decreased the heparin dose requirement in the post-hoc analysis [23]. A recent study from a single institution has shown that the manufacturer’s dosing guidelines for antithrombin (human) are inadequate to achieve recommended AT levels in neonates on ECMO [24]. The AT administration guidelines are given in Table 1 and Table 2.

In heparinized patients, when feasible, subcutaneous and intramuscular injections, nasogastric tube and Foley catheter insertion, rectal temperatures, heel sticks, and non-invasive cuff blood pressure measurements should be avoided.

## 6. Heparin Anticoagulation Monitoring

*Activated Clotting Time (ACT)*—A point of care coagulation test ACT has been used for a long time during ECMO therapy. It is a simple test that measures the time of whole blood to form a fibrin clot at the bedside by exposing the sample of an activator (Kaolin or Diatomaceous earth). It is an important low-cost bedside test available to monitor anticoagulation with UFH [25]. Although the current ELSO guidelines do not specify a number [12], the suggested ACT range during the ECMO support is 180–220 s. Poor technique in sample collection, hypothermia, hemodilution, presence of exogenously added anticoagulants, thrombocytopenia or platelet dysfunction, and factor deficiencies will interfere with the test results. Compared to the gold standard, the plasma heparin level assay (anti-factor Xa activity) ACT has a poor correlation [26,27]. In children, physiologically low AT levels than adults result in impaired response to heparin, so using ACT only to guide heparin dosing causes inadequate anticoagulation [28,29,30]. In addition, ACT results vary based on the device used [31]. Due to these potential caveats, some centers gradually replaced ACT; even when it is used, it is supplemented by the more definitive tests like the anti-factor Xa assay [32].
*Activated Partial Thromboplastin Time (aPTT)*—Activated partial thromboplastin time is a plasma test that measures the hemostasis in the absence of cellular components, especially platelets. A blood specimen should not be drawn through the indwelling catheter with heparin infusion to avoid possible heparin contamination, and the specimen to be transported and stored at 2 °C to 4 °C. The usual aPTT range during ECMO is 60–80 s [12]. In children compared to adults, aPTT poorly correlates with anti-factor Xa levels [33,34]. Adult patients on ECMO with aPTTs of 1.5–2.5 times normal values have shown a good correlation with UFH concentrations of 0.2–0.4 U/mL [35]. APTT is frequently prolonged in children during ECMO despite an age-appropriate UFH dose administration due to developmental differences in hemostasis [36]. Multiple biological variables can affect the heparin monitoring by the aPTT. The individual center-specific laboratory needs to determine the appropriate therapeutic range.
*Anti-factor Xa assay*—Recently, the anti-factor Xa assay has become a gold standard test to monitor UFH and low-molecular-weight heparin (LMWH) management even outside the ECMO support [37]. The principle of this essay depends on the heparin’s ability to inhibit factor Xa or thrombin by AT. Therefore, precautions similar to aPTT measurement should be taken to draw the blood sample. In this assay, heparin neutralization is done using protamine sulfate or polybrene. The anti-factor Xa assay estimates UFH activity and does not measure UFH concentration [38]. Recommended therapeutic anti-factor Xa levels are between 0.3–0.7 IU/mL and correlate to a heparin level of 0.2–0.4 U/mL [19]. Many automated coagulation analyzers can estimate heparin activity. Except for AT deficiency, it is not affected by acute phase reactants like factor VIII and fibrinogen or factor deficiencies. In addition, the anti-factor Xa assay eliminates the need to establish aPTT therapeutic range. This assay is also useful in patients with baseline elevated aPTT. However, the anti-factor Xa assay requires prompt sample processing to avoid platelet factor 4 mediated heparin neutralization. It is more expensive than the aPTT and it underestimates heparin activity in the presence of significant AT deficiency. In addition, the test is less sensitive in the presence of free hemoglobin, high bilirubin, and high triglyceride levels [39].

Anti-factor Xa assay poorly correlated with ACT assay in children and adults undergoing cardiopulmonary bypass (CPB) for cardiac surgery [40,41]. Compared to the ACT assay, the anti-factor Xa assay measures only the heparin effect on AT, whereas the ACT assay measures the whole blood clotting time in the presence of heparin and other patient-related coagulation factors [40].

*Viscoelastic Hemostatic Assays (VHAs*)—Thromboelastography (TEG) and rotational thromboelastometry (ROTEM) are commonly used VHAs. They analyze the viscoelastic properties of blood clot formation on a whole blood sample and graphically display all stages of the development and resolution of the clot. TEG/ROTEM reflects the global hemostatic function, including coagulation cascade integrity, platelet function, and fibrinolysis [42]. Therefore, VHAs are very useful for patients on ECMO as they can have multiple etiologies for the underlying coagulation abnormalities [43]. In addition, they are particularly beneficial and recommended in patients with bleeding during ECMO, cardiac surgery, and trauma [44,45,46,47]. Although VHAs are real-time tests to assess the whole blood coagulation status, they measure hemostasis in vitro, so the results need to be interpreted in relation to the patient clinical condition. Moreover, blood collection site, sample processing (native vs. citrate sample), patient age, and gender should be considered when interpreting the results, as these factors can affect the results [48]. ECMO anticoagulation laboratory monitoring schedule is shown in Table 3 based on recent ELSO guidelines [12]. However, laboratory tests and frequency of monitoring widely vary based on patient clinical status and among the ECMO centers.

## 7. Heparin-INDUCED Thrombocytopenia (HIT)

HIT is an immune-mediated reaction that may occur in patients on heparin therapy for at least five days [49]. HIT is a serious adverse effect, and these patients present with a greater than 50% drop in the platelet count. HIT is mediated by antibodies against heparin/platelet factor 4 (H/PF4) complexes. Interaction of these antibodies with platelet Fc-receptor IIA activates platelets and results in thrombocytopenia and thrombosis. In addition, endothelial cell activation of H/PF4 complexes increases tissue factor expression and thrombin production. Incidence of HIT in adults varies from 1 to 5%, and limited data suggest a similar incidence in children. It is more prevalent in patients exposed to UFH than LMWH. Incidence is highest with therapeutic doses compared to prophylactic doses and when the heparin is given by the intravenous route. Patients with HIT develop arterial or venous thrombosis and have a high risk of mortality [50].

HIT is a clinical diagnosis. Highly sensitive ELISA for anti-H/PF4 antibody and highly specific serotonin release functional assays are confirmatory tests. Heparin exposure in all forms should be discontinued once the HIT diagnosis is made and investigated for the presence of thrombosis. For thrombosis, direct thrombin inhibitors like argatroban should be used for anticoagulation [51]. The anticoagulation duration with alternative anticoagulants varies depending on the presence or absence of thrombosis and the length of the ECMO support [52].

## 8. Heparin Resistance and Antithrombin Replacement during ECMO

Heparin resistance should be suspected when an increased heparin dose is required to achieve the desired anticoagulation effect. The literature describes heparin resistance as UFH doses greater than 35,000 units/day in adults, while such cut-offs are poorly described in children and infants [53]. AT deficiency is the most common cause of heparin resistance during the ECMO. The usual range of AT activity level in adults is between 80–120%, and there is no established baseline AT levels in children. Although optimal AT dosing is not defined in ECMO patients, recently, there has been a significant rise in AT replacement. However, the AT replacement efficacy and safety during ECMO support need to be evaluated [54].

## 9. Fibrinolysis and Management

Along with the coagulation cascade, the fibrinolytic pathway is also activated by vascular endothelial injury (see Figure 1). Fibrinolysis dissolves the stable fibrin clot and helps vessel patency and remodel the damaged vascular endothelium. The initial step of fibrinolysis involves the conversion of tissue plasminogen (tPA) to active plasmin, which dissolves the fibrin clots and produces fibrin degrading or fibrin split products (FDP or FSP). An elevated FDP level in the plasma indicates an activated fibrinolysis pathway. Fibrinolytic pathway activation should be suspected in a bleeding patient with appropriate levels of platelets and coagulation factors. Epsilon aminocaproic acid (EACA/Aminocaproic acid), tranexamic acid (TXA), and aprotinin all inhibit the conversion of tPA to plasmin resulting in fibrin clot formation and controlling the bleeding [55,56,57]. EACA and TXA have been used successfully in many centers to manage significant surgical site bleeding [56,57,58]. A recent study reported a significant decrease in bleeding, red cell transfusions, and improved coagulation profile among pediatric cardiac patients during ECMO support [58]. Aprotinin use in patients with cardiac surgery has shown to be associated with an increased risk of renal dysfunction [59]. Before administering aminocaproic acid, assess the fibrinolysis activity by doing a VHA. During fibrinolysis, the typical ROTEM findings show an increased ML (Maximal Lysis at 30 min) activity in INTEM, EXTEM, and APTEM tests. At our institution, EACA is used for the management of fibrinolysis when indicated. The suggested dose of EACA is 50–100 mg/kg bolus over one hour, followed by a continuous drip of 10–40 mg/kg/h. This drug will help attain hemostasis in the anticoagulated ECMO patient. It will also precipitate clotting in the extracorporeal circuitry. Close monitoring for circuit clotting is suggested with antifibrinolytic usage during ECMO. Aminocaproic acid is indicated only for patients at high risk of bleeding. Aminocaproic acid therapy should be titrated to keep FDP levels 10–40 mcg/mL and ACT 180–220 s (Table 4).

## 10. Excessive Bleeding Management during ECMO

Patients on ECMO are at risk of bleeding, which is a significant cause of morbidity and mortality. Excessive platelet and coagulation factors consumption in the circuit during ECMO and anticoagulation increases the chances of bleeding. Young children receiving ECMO are known to have lower levels of coagulation factors at baseline [60]. Most patients requiring ECMO are prone to have some degree of disseminated intravascular coagulation (DIC) due to their underlying severe illness. Surgical site bleeding is reported in 20–29.3% of ECMO patients with cardiac surgery [47]. Central nervous system bleeding is a potentially fatal complication that occurs in 3–6% of patients on ECMO, with neonates having the highest risk. Cardiovascular instability, hypoxemia, acidosis, prematurity, coagulopathy, jugular, and carotid artery ligation are known risk factors for intraventricular hemorrhage [61].

Significant blood product replacement is necessary during ECMO to decrease the risk of bleeding complications and treat clinical bleeding in a patient on ECMO. There is limited literature to guide blood product transfusions in ECMO patients. The current ELSO guidelines recommend maintaining hematocrit >40%; however, in children, there is no defined safer low hemoglobin threshold [12]. A restrictive transfusion strategy is favored among adult ECMO patients [62]. Similarly, platelet transfusion practice is widely variable among pediatric ECMO centers, with a median platelet count range of 50,000 to 200,000 × 10^9^/L [8]. A subgroup analysis of two large point prevalence studies involving pediatric ECMO patients reported a median platelet count threshold of 70,000 × 10^9^/L and a median international normalized ratio (INR) of 1.4 for the platelet and fresh frozen plasma transfusions, respectively [63]. In neonates, it is recommended to maintain a platelet count above 100,000 cells/mm^3^. Ideally, blood products need to be given directly to the patient via a peripheral intravenous line. If it is not feasible, then the blood products may be given in the circuit post centrifugal pump. To control oozing at surgical sites using thrombin-based fibrin glue or cryoprecipitate-topical thrombin glue is efficacious. Fibrinolytic pathway inhibitors like aminocaproic acid, tranexamic acid, and aprotinin can be used to manage surgical site bleeding [55,56,57]. Various blood product transfusion parameters and guidelines are outlined in Table 5.

For intractable surgical bleeding recombinant factor VIIa (rVIIa) has been used successfully to control the bleeding [64,65]. rVIIa promotes thrombin production; its usual dose is 40–90 mcg/kg. There was a significant reduction in chest output and reduced red blood cell transfusions following rVIIa use [64]. However, increased circuit clots and cases of fatal thrombosis have been reported with rVIIa administration in patients with ECMO [66,67,68].

## 11. Non-Heparin Anticoagulants

### Direct Thrombin Inhibitors

Direct thrombin inhibitors (DTIs) are relatively new classes of anticoagulants that work independently of AT levels [69]. Their independent action of AT makes them suitable to use in young children. Compared to heparin, these agents have more predictable pharmacokinetics and a higher thrombin production inhibition [70]. In addition, DTIs affect both bound and free thrombin. Argatroban, bivalirudin, and lepirudin are some of the agents used in ECMO, especially in patients with HIT, heparin resistance, and patients with significant clot burden while on heparin therapy [51,71]. Bivalirudin is the most frequently used DTI as an alternative to heparin during ECMO support due to its favorable pharmacokinetic profile. In contrast to argatroban and lepirudin, bivalirudin has the shortest half-life, 25 min, and is predominantly metabolized via proteolytic degradation and partly by renal clearance [72]. The dosing, use of loading dose, and continuous infusion of bivalirudin are variable in children ranging from 0.04 to 0.48 mg/kg/h [72]. Higher doses of bivalirudin are needed in children on renal replacement therapy [72]. Blood stagnation must be avoided in the ECMO circuit while on bivalirudin due to its short half-life and proteolysis, which can lead to circuit thrombosis [73]. Although aPTT is not a validated laboratory assay to monitor DTI therapy, the dosing is adjusted by maintaining aPTT ratios of 1.5–2.5. Available data for bivalirudin use suggests no increase in side effect profile and a modest decrease in the odds ratio of mortality in adult patients [74]. While there are presently no antidotes available, discontinuation is the primary measure in case of supratherapeutic anticoagulation due to their short half-lives. Bivalirudin is shown to be removed by continuous renal replacement therapy and plasmapheresis [75]. Currently, there is limited data available regarding DTI safety and efficacy among children receiving ECMO.

## 12. Antiplatelet Therapy

During the ECMO, blood exposure to the artificial biomaterial causes platelet activation and aggregation, leading to increased thrombotic risk. Aspirin is the commonly used antiplatelet agent, which acts through noncompetitive inhibition of platelet cyclooxygenase-1 and thromboxane A2 production. The addition of aspirin may reduce the ECMO circuit and patient clot burden. However, there is no data available on children supported with ECMO. The evidence for using aspirin is limited to a few adult patients receiving ECMO [76].

## 13. Knowledge Gap and Future Perspectives

Currently, there is no ideal anticoagulant agent to make the ECMO circuit as non-thrombogenic as vascular endothelium. An endothelial cell inhibits platelet adhesion and activation by producing nitric oxide (NO) and prostacyclin [77]. Incorporating the NO and prostacyclin into the ECMO circuit showed reduced platelet consumption in studies [78,79]. Heparin bonded circuits are used in many centers to make the circuits more biocompatible and limit anticoagulation usage during cardiopulmonary bypass and ECMO. Studies showed a reduced platelet activation, fibrinolysis, inflammatory response, and decreased blood loss and blood product usage with heparin-coated circuits; however, these effects last a very short time, limiting their use in ECMO [80,81]. Some centers utilize a multi-system therapy protocol using dipyridamole, aspirin, aprotinin, and pentoxifylline. This protocol showed a reduction in bleeding complications and survival benefits compared to the control patients treated with heparin and ACT monitoring alone for anticoagulation [82]. However, these anticoagulant therapies are not standardized in pediatric ECMO patients. Further studies are needed to improve anticoagulation management in children during ECMO therapy. Due to age-related developmental hemostatic changes, children are at higher risk of bleeding. In addition, significant variation in clinical practice and lack of standardized anticoagulation management warrants further studies to determine the pharmacokinetics and pharmacodynamics, as well as the safety and efficacy of these therapies.

## 14. Limitations

In this manuscript, we described the pathophysiology of coagulation during ECMO support, reviewed the literature on current and newer anticoagulant agents, and outlined various diagnostic tests used for anticoagulation monitoring. However, we cannot suggest the standard anticoagulation therapy and monitoring due to significant practice variability in children receiving ECMO support and the lack of robust evidence for any one particular practice. In addition, the laboratory monitoring frequency and the parameters cut-off values mentioned in this manuscript may vary among various pediatric ECMO centers.

## 15. Conclusions

Anticoagulation during pediatric ECMO support is challenging due to complex interactions between the patient and the ECMO circuit. In addition, children are at higher risk of bleeding than adults because of age-related developmental hemostatic changes. Although there is no ideal anticoagulant, UFH is the most commonly used agent for anticoagulation. DTIs are the alternative anticoagulants with predictable pharmacokinetics; however, their safety and efficacy data is currently limited in children. Lack of standardized anticoagulation protocol applicable to all ECMO centers, resulting in wide clinical practice variability. Advancement in ECMO circuit designs and materials and gaining more experience with newer anticoagulants may decrease the bleeding and thrombosis risk and ultimately improve outcomes in children receiving ECMO support.

## Figures and Tables

**Table 1:** Human and recombinant Antithrombin (AT) indication, dose, complications & monitoring parameters *.

**Indications**
1. AT deficiency 2. Heparin resistance
**Recombinant AT Dose**
**Loading dose** (Units) $\frac{\left[100-Baseline\;AT\;activity\right]\;\times \;\left[Body\;Weight\;\left(\mathrm{Kg}\right)\right]}{2.3}$
Loading Dose is given over 15 min immediately followed by a continuous infusion of the maintenance dose
**Maintenance dose** (Units/h) $\frac{\left[100-Baseline\;AT\;activity\right]\;\times \;\left[Body\;Weight\;\mathrm{in}\;\left(\mathrm{Kg}\right)\right]}{10.2}$
**Human AT dose**
**Loading dose** (Units) $\frac{\left[120-Baseline\;AT\;activity\right]\;\times \;\left[Body\;Weight\;\left(\mathrm{Kg}\right)\right]}{1.4}$
**Maintenance dose** (q 24 h) Loading Dose × 0.6
**Complications**
1. Bleeding 2. Anaphylaxis 3. Hematuria
**Monitoring parameters**
AT levels q 12 h Unfractionated heparin assay q 6 h

* Based on manufacturer’s recommendations.

**Table 2:** Recombinant Antithrombin (AT) dose adjustment *.

**Initial Monitoring Time**	**AT Level**	**Dose Adjustment**	**Recheck AT Level**
2 h after initiation of treatment	<80%	Increase 30%	2 h after each dose adjustment
	80–100%	None	6 h after initiation of treatment or dose adjustment
	>100%	Decrease 20%	2 h after each dose adjustment

* Based on manufacturer’s recommendations.

**Table 3:** Extracorporeal membrane oxygenation (ECMO) anticoagulation laboratory monitoring protocol **.

Laboratory Tests	Frequency	Target Range
ACT	Every 1 h for first six hours of ECMO, then every 2 h if stable.	Range 180–220 s. If there is excessive bleeding decreases the target as per MD.
PT/aPTT/INR	Q 6–12 h	PT 10–13 s PTT 1.5–2.5 times normal (60–80 s) PT/INR normal or close to normal <1.5
Heparin Assay Unfractionated (Anti-Xa)	Q 6–12 h	0.3–0.7 IU/mL
Antithrombin (AT)	Q 12 h, once level is therapeutic and stable: Q 24 h.	50–80% * Start recombinant AT and follow protocol if low
Fibrinogen/FDP	Q 12–24 h	Fibrinogen > 150 mg/dL (bleeding patient) >100 mg/dL (nonbleeding patient) FDP 10–40
Platelet Count	Q 6 h first 48 h, then Q 12 h.	50,000–100,000 × 10^9^/L unless VHA indicates need to give >100,000 × 10^9^/L if bleeding.
Rotem^®^/TEG^®^	Daily, PRN for severe bleeding or thrombosis(Compare with Heparinase sample)	Clotting Time (CT) Normal: 5–10 min
		Clot formation time (CFT) Normal: 1–3 min Target: 3× CFT of Heparinase sample
		Alpha angle (α) Normal: 53–72 degrees
		Max clot firmness/Clot strength (MCF) Normal: 50–70 mm Target: >50 in bleeding
		Lysis Index 30min after CT (LY30) Normal: 0–7%

** As per ELSO guidelines. * Based on manufacturer’s recommendations. ACT = Activated Clotting Time; aPTT = Activated Partial Thrmboplastin Time; FDP = Fibrinogen Degradation Products; INR = Intranational Normalized Ratio; PT = Prothrombin Time; ROTEM = Rotational Thromboelastometry; TEG = Thromboelastography; VHA = Viscoelastic Hemostatic Assay.

**Table 4:** Aminocaproic acid indications, dose, complications & monitoring parameters.

**Indications**
1. In patients at risk for bleeding including all post-operative patients 2. In patients with significant bleeding with normal platelets and coagulation factors
**Dose**
The dose is 50–100 mg/kg bolus over one hour followed by continuous drip of 10–40 mg/kg/h. (Maximum dose 30 gm/day)
**Complications**
Clots in the extracorporeal circuit Anaphylaxis
**Monitoring parameters**
ACT 180–220 s FDP 10–40 mcg/mL Fibrinogen > 200 mg/dL Platelets > 150,000/mL

**Table 5:** Blood product transfusion guidelines during extracorporeal membrane oxygenation **.

Parameter	Goal	Guideline
PRBC’s	Hemoglobin 70–90 gm/L	PRBC’s 10 mL/kg (maximum 2 units)
Platelets	>80,000 >100,000 × 10^9^/L (in bleeding patients)	Platelets 10 mL/kg (maximum 2 units)
FFP	INR < 1.5 (bleeding patient) INR < 3 (nonbleeding patient)	FFP 10 mL/kg (maximum 2 units)
Cryoprecipitate	Fibrinogen > 1.5 gm/L (bleeding patient) >1 gm/L(nonbleeding patient)	Number of units = [(200-fibrinogen) (kg)] ÷ 200 1 unit/5 kg (maximum 6 units)

** Based ELSO guidelines. FFP = Fresh Frozen Plasma; PRBC = Packed Red Blood Cells;.
